# Multiple *Drosophila* Tracking System with Heading Direction

**DOI:** 10.3390/s17010096

**Published:** 2017-01-05

**Authors:** Pudith Sirigrivatanawong, Shogo Arai, Vladimiros Thoma, Koichi Hashimoto

**Affiliations:** 1 Graduate School of Information Sciences, Tohoku University, Aramaki Aza Aoba 6-6-01, Aoba-Ku, Sendai 980-8579, Japan; koichi@m.tohoku.ac.jp; 2 Graduate School of Engineering, Tohoku University, Aramaki Aza Aoba 6-6-01, Aoba-Ku, Sendai 980-8579, Japan; arai@irs.mech.tohoku.ac.jp; 3 Graduate School of Life Sciences, Tohoku University, Katahira 2-1-1, Miyagi, Sendai 980-8577, Japan; thoma_vlad@hotmail.com

**Keywords:** Drosophila, tracking, Kalman filter, machine vision

## Abstract

Machine vision systems have been widely used for image analysis, especially that which is beyond human ability. In biology, studies of behavior help scientists to understand the relationship between sensory stimuli and animal responses. This typically requires the analysis and quantification of animal locomotion. In our work, we focus on the analysis of the locomotion of the fruit fly Drosophila melanogaster, a widely used model organism in biological research. Our system consists of two components: fly detection and tracking. Our system provides the ability to extract a group of flies as the objects of concern and furthermore determines the heading direction of each fly. As each fly moves, the system states are refined with a Kalman filter to obtain the optimal estimation. For the tracking step, combining information such as position and heading direction with assignment algorithms gives a successful tracking result. The use of heading direction increases the system efficiency when dealing with identity loss and flies swapping situations. The system can also operate with a variety of videos with different light intensities.

## 1. Introduction

The study of model organisms provides an understanding of how biological processes work, which benefits humans in terms of medical knowledge and understanding of other biological behaviors [1]. One of the popular model organisms that has been heavily used in research is Drosophila melanogaster, or generally known as the common fruit fly. With this model organism, a variety of genetic methods can be used to manipulate the activity of specific neurons in freely moving and behaving animals [2].

A common way to analyze behavior is to categorize and quantify animal movements. Locomotion observation of insects such as bees [3], flies [4], and ants [5], using machine vision systems has been used for the analysis. In our work, we focus on using a machine vision system to track freely walking Drosophila melanogaster in a circular arena. Considering the fact that it has a small size and it can sometimes jump; this causes great difficulties for motion observation.

Dealing with only a single fly, its information can be provided in detail and the motion observation can be easily implemented, but the motion related to interactive behavior among a group of multiple flies will be absent. There are some researchers working on the behavior of a single fly. Zabara et al. study the takeoff dynamic of a single Drosophila [6,7]. A study of freely flying Drosophila is done by [8]. Fan et al. study the behavior of Drosophila using two flies [9]. However, it takes time until they show interactive behavior.

A machine vision system with the presence of multiple Drosophila fulfills the motion observation in the way that it can provide observation on the behaviors of interacting flies that cannot be observed from the single fly scenario. More flies provide more data and in addition, sexual behavior can also be observed. However, dealing with multiple flies is challenging to the system since the system has to be able to track each individual fly. This means that the system has to give each fly an identifier and maintain its identifier throughout the observation period.

This paper introduces a machine vision system that can detect and track multiple Drosophila in a video input. The overall process of this system is divided into three main parts: foreground detection, posture modeling, and tracking.

The rest of this paper is organized as follows: Section 2 shows reviews of related work. Materials and methods, provided in Section 3, consists of our work. Section 4 presents the results and a discussion with an example of using our tracking system. Finally, a conclusion to our work is given in Section 5.

## 2. Related Work

To achieve a successful result from a video input, firstly, the system needs to be able to distinguish Drosophila from the background. Based on the fact that Drosophila move while other objects stay in the same frame, this gives us an opportunity to use the mode value of intensity to extract background information from the image scene. Background extraction with a moving foreground is done by [10]. Once the background has been extracted, background subtraction [11] is then performed to split up Drosophila and other objects. This not only improves the accuracy of Drosophila detection but also prevents the system from wasting processing time on other objects beside the fly. The background subtraction example can be seen from [12,13] with an application of video surveillance and monitoring. Other applications using the background subtraction include optical motion capture [14], human computer interaction [15], and manufacturing industry [16].

After the Drosophila have been isolated from the background, the resulting image needs to be polished in order to remove the remaining noises. A polishing process can be accomplished by normal binary thresholding or by applying opening mathematical morphology [17]. In our system, a Laplacian of Gaussian filter [18] is applied to the resulting image to provide smooth and noiseless blobs result. Next, ellipse fitting is performed at each blob and the major axis—an axis representing the widest part of the ellipse—is calculated. A region-based method for ellipse fitting is discussed in [19].

To the best of the authors’ knowledge, there are many off-the-shelf tracking software packages available. One of the most well-known Drosophila tracking programs is C-Trax [20]. It is an open-source software that is freely available for estimating the positions and orientations of walking flies. The program comes with the ability to automatically classify the flies’ behaviors. In addition to C-Trax, another system that is well-known for measuring animal behavior is JAABA [21]. JAABA uses a machine learning-based system to compute behavior of animals together with the input trajectory from other tracking programs. idTracker [22] provides a tracking system that can be used with a wide range of animals including zebrafish, medaka fish, mice, flies, and ants. EthoVision XT is suitable for a wide range of set-ups but it has a weak point when dealing with a large number of animals with interactions.

In our work, we present a multiple Drosophila tracking system that works without needing any modification of the test subjects. Our system is able to detect and track each individual fly in the scene while providing an accurate result obtained from using a Kalman filter. In addition, it uses a combination of prediction from the Kalman filter and the closest neighbor principle in order to achieve better results in tracking selection. Heading direction is also used for confirmation of fly pairing between two consecutive frames. The proposed system is a platform for future development.

## 3. Materials and Methods

For an input video in a range of interested frames, foreground detection and posture modeling are processed first for all frames. Next, position and heading direction obtained from these two steps will be used in the tracking step.

### 3.1. Foreground Detection

**Figure 1:**
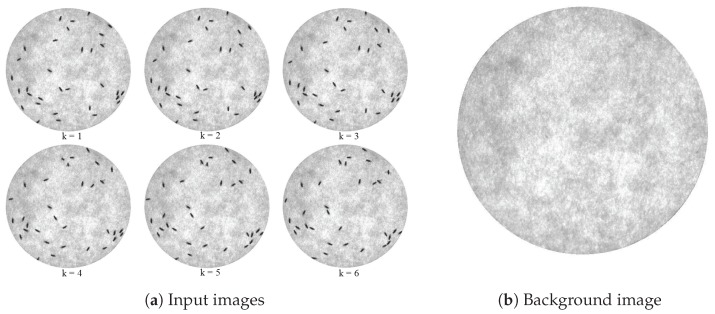
(**a**) Input images and (**b**) extracted background image. The input image contains six consecutive frames of a circular arena with 32 *Drosophila* as the black ovals inside. The background image was generated by using a number of frames from the same video file as the input images.

At each time step, an input image is taken from image sequences from an input video and converted into a gray scale image. The background image is extracted by considering only the static part of the image sequences. In this way, the pixel value that frequently appears in the image sequences is treated as background while the one with less frequency will be considered as foreground. In order to determine the value that has the highest frequency at each pixel, a vector containing pixel values from the image sequences at the same pixel position is created. Mode calculation on the vector is performed and the most frequent value is then obtained. This process is repeated for every pixel position of the image sequences, thus the background is created. A total of 100 images are randomly selected from the image sequences and are used in the background extraction step instead of the whole image sequences. Figure 1 shows an example of six consecutive images of Drosophila (Figure 1a) and extracted background images (Figure 1b).

In some cases, the input video file suffers from the varying light in the scene due to the non-perfect experimental setup. The varying light affects the ability of detection and may result in missed detections. Therefore, intensity adjustment is performed, using the background image as a reference for intensity shifting. The intensity shifting is done by two steps: first, calculating the intensity mean values of both the background image and the current input image, second, updating the intensity of every pixel in the input image by adding the difference between the intensity mean values of the background image and the current input image to all pixels.

**Figure 2:**
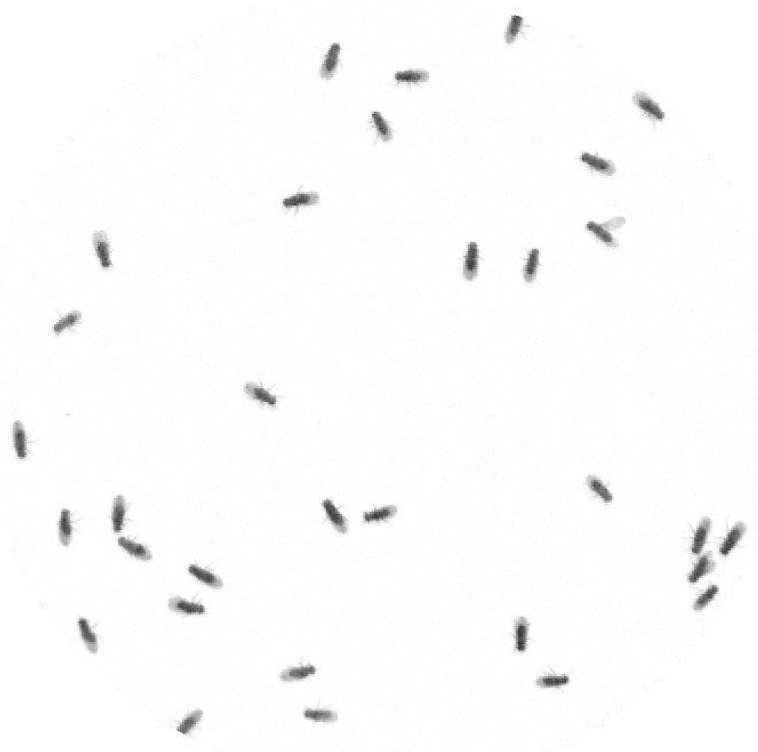
Resulting image from background subtraction followed by intensity reversal. The static arena part that belongs to the background was deleted. Only moving flies’ body and their wings remain in the resulting image.

The foreground is detected by performing background subtraction with the current input image. In this step, some noises may appear in the resulting image. Figure 2 illustrates the resulting image after performing background subtraction followed by intensity reversal. The static arena part is deleted and only Drosophila are left in the scene.

**Figure 3:**
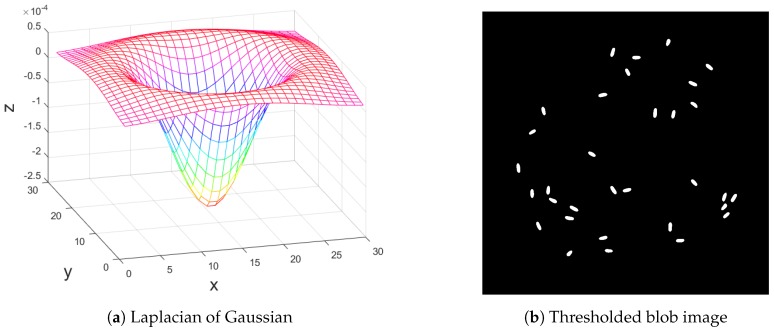
(**a**) Laplacian of Gaussian as a kernel in 3-D view and (**b**) thresholded blob image. The kernel is used for convolution with the resulting image from background subtraction. Thresholding is applied to the product of the convolution, resulting in the thresholded blob image.

Next, a blob filter is applied to the image. The blob filter uses Laplacian of Gaussian as a kernel matrix for convolution with the image. Laplacian of Gaussian appearance is shown in Figure 3a. According to the fact that the fly’s body in Figure 2 is dark, the Laplacian of Gaussian makes the body white. The convolution makes bright pixels dark and dark pixels white. In this way, the fly’s body will appear as a white blob. Next, a proper user-defined threshold is applied to the blob image in order to get rid of some noises. In this thresholding step, the unwanted part will be deleted from the scene and the wanted part will be preserved in the scene. Here, this is not binary thresholding, therefore every pixel in every blob still possesses detailed intensity values. Figure 3b shows a resulting image after applying the blob filter and thresholding.

A two-dimensional Laplacian of Gaussian function is written as

(1) $$\begin{array}{c} LoG\left(x,y\right)=-\frac{1}{\pi \sigma^{4}}\left(1-\frac{x^{2}+y^{2}}{2\sigma^{2}}\right)e^{-\frac{x^{2}+y^{2}}{2\sigma^{2}}} \end{array}$$

where *x* and *y* are Cartesian coordinates. Standard deviation is user-defined and is shown as *σ*. The value of standard deviation used in Figure 3a is σ=6.

In the case of multiple foreground objects, sometimes a group is detected as only one blob, meaning that one object is detected instead of multiple objects. This results in an error of detected positions and will decrease the tracking efficiency in later steps. This problem takes place when some foreground objects locate very close to one another. To overcome such a problem, blob analysis is introduced.

The blob analysis process starts by first calculating the parameters of every blob in the blob image such as area, bounding box, and center of the blob. Second, average area of all the blobs is determined and considered as an expected area of one object. Third, at each blob, the expected number of objects is calculated by dividing the blob area by the expected area. If the expected number of objects is one, then that blob is considered to be correct, and no further modification is necessary. On the other hand, an expected number of objects larger than one means that multiple objects are merging together and detected as a single blob.

**Figure 4:**
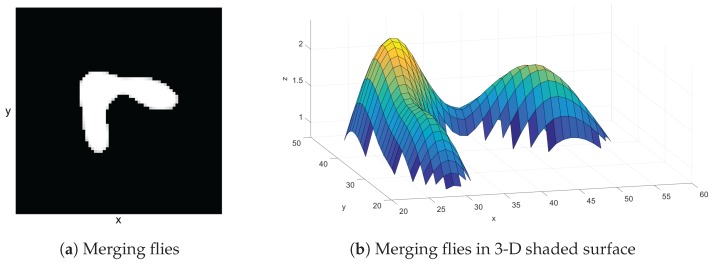
(**a**) Merging flies in a 2-D scene and (**b**) in a 3-D shaded surface. The merging flies appear as a single white area. By observing the merging flies in a 3-D shaded surface, this single area seems to contain two flies since there are obviously two mountains in the image.

**Figure 5:**
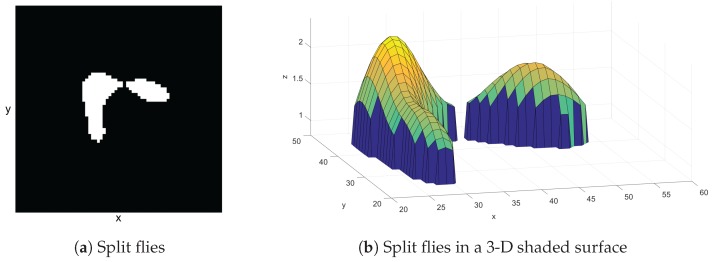
(**a**) Split flies in a 2-D scene and (**b**) in a 3-D shaded surface. The merging flies blob from Figure 4 was split by varying threshold values until the blob is split into an expected number of flies.

In this case, the process of extracting objects that are merged is done by varying a threshold value and redoing the thresholding step in that specific blob region. The threshold value is increased by one step value per iteration and there is a maximum number of iteration steps. The process of threshold varying stops when the number of blobs obtained from applying the increased threshold value equals the expected number of objects or the iteration reaches its maximum. Measurements of parameters such as area, bounding box, and center of the blob are also performed at the newly extracted blobs. Figure 4 shows what merging flies look like in a blob image (Figure 4a) and merging flies on a 3-D shaded surface (Figure 4b). The threshold level is not high enough to separate the flies. The result, after the system splits the merging flies, is shown in Figure 5. Note that the threshold level was increased to be just enough for separation of the blob. The splitting of merging flies only works when the flies are not overlapping too much. If they are overlapping too much, the varying of the threshold will reach its maximum without reaching the expected number. This makes the program detect them as a single fly. However, in our system, multiple identities can be assigned to the same detection.

### 3.2. Posture Modeling

The posture of a Drosophila includes its position and heading direction. The position of each Drosophila is the center of its blob. Note that this has already been done in the blob analysis step. The center of a blob can be calculated by

(2) $$\begin{array}{c} \bar{x}=\frac{1}{N}\sum_{i\in I}x_{i} \end{array}$$

(3) $$\begin{array}{c} \bar{y}=\frac{1}{N}\sum_{i\in I}y_{i} \end{array}$$

where $\bar{x}$ and $\bar{y}$ are the center in the *x* and *y* direction, respectively. The number of connected pixels that form the blob is *N*. Set I contains all indexes of those pixels.

**Figure 6:**
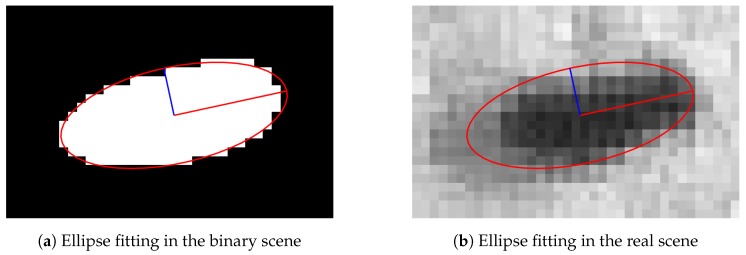
(**a**) Ellipse fitting result in the binary scene and (**b**) in the real scene. The ellipse fits the filtered data in the binary scene. The major and the minor axes are illustrated as the red and the blue lines, respectively. According to the real scene, the head of the fly and wings’ position both lie on the main axis. Being able to determine the position of the wings implies the position of the head.

To obtain the heading direction, the orientation of each object is first approximated since the heading direction lies on the orientation vector. This is done by performing ellipse approximation to each blob and then calculating the major axis of the best-fit ellipse. Once the orientation of every blob is determined, measurement of heading direction is then performed. The ellipse approximation can be done by following Equations (4)–(10). Equations (4)–(6) calculate the normalized second central moment for the region. The term $\frac{1}{12}$ is the normalized second central moment of a pixel with unit length.

(4) $$\begin{array}{c} u_{xx}=\frac{1}{N}\sum_{i\in I}{\left(x_{i}-\bar{x}\right)}^{2}+\frac{1}{12} \end{array}$$

(5) $$\begin{array}{c} u_{yy}=\frac{1}{N}\sum_{i\in I}{\left(y_{i}-\bar{y}\right)}^{2}+\frac{1}{12} \end{array}$$

(6) $$\begin{array}{c} u_{xy}=\frac{1}{N}\sum_{i\in I}\left(x_{i}-\bar{x}\right)\left(y_{i}-\bar{y}\right)+\frac{1}{12} \end{array}$$

(7) $$\begin{array}{c} \Delta =\sqrt{{\left(u_{xx}-u_{yy}\right)}^{2}+4u_{xy}^{2}} \end{array}$$

(8) $$\begin{array}{c} a=\sqrt{2(u_{xx}+u_{yy}+\Delta )} \end{array}$$

(9) $$\begin{array}{c} b=\sqrt{2(u_{xx}+u_{yy}-\Delta )} \end{array}$$

(10) $$\theta =\left\{\begin{array}{cc} \arctan(\frac{u_{yy}-u_{xx}+\Delta }{2u_{xy}}) & \mathrm{if}\;u_{yy}>u_{xx}\\ \arctan(\frac{2u_{xy}}{u_{xx}-u_{yy}+\Delta }) & \mathrm{if}\;u_{xx}>u_{yy}\\ 0 & \mathrm{if}\;u_{xy}=0\;\mathrm{and}\;u_{xx}-u_{yy}+\Delta =0 \end{array}\right.$$

where *a* and *b* are major and minor axes, respectively. The orientation is represented by *θ*. Figure 6 shows an example of ellipse fitting to a blob (Figure 6a). Figure 6b shows the ellipse with the real scene. The major axis is represented by a red straight line while the minor axis is represented by a blue straight line.

According to the orientation of a blob, there are two choices of direction: one pointing in the direction of the head and another pointing in the reverse direction. The problem is how to determine which direction from the two choices is the correct one for heading direction. One method of detecting the heading direction is to detect the positions of the wings, because they are always located at the opposite direction to the head. Our system uses the idea that wings have an intermediate intensity between the fly’s body and background to distinguish them. Wing detection of a blob is done by deploying two search points to that blob along with applying a specific range of threshold couples to all the pixels in the bounding box of the blob. The two search points are decided from the points located at the end of the blob’s major axis, which are normally the location of the wings. A threshold couple is introduced to extract the wing parts. For each threshold couple, two threshold values are presented, floor value and ceiling value. When the threshold couple is applied to an image, the pixel value that is below the floor value and is above the ceiling value will be assigned with zero. On the other hand, the one with the value between the floor and ceiling values will be assigned with one. The system gives a score to each of the two search points and the score is obtained by collecting the assigned values of all pixels located around each search point within a specific radius. The search point that has a higher score, that is above a specific value, will be considered as the representation of wing position. The system also counts a score for the pixels outside the search area, noted as environment pixels. For a search point having a high score with a high score of environment, the search point will be rejected from being the representative and the score of that point will not be counted. In this case, what the search point found is not the wing pixel but the environment pixel. The process runs by alternating various threshold couples and collecting the score of those search points for a certain iteration step. Finally, the heading direction for each blob is obtained by calculating a direction from the blob centroid to the search point that has a higher score. These position and heading directions will be used in the tracking step later on. The algorithm used for determining the heading direction is shown in Algorithm 1.

**Figure 7:**
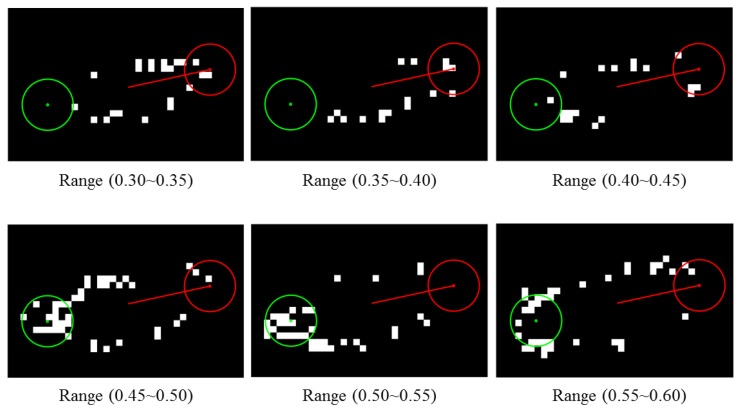
Filtered image at different threshold range levels. Two search points illustrated as red and green are located at the end of the major axis. A number of threshold ranges are applied to a blob resulting in white pixels. Each search point collects a score by counting a number of white pixels located within its range. The higher the number of white pixels that a search point found, the higher the probability of that point to contain wings. In this figure, wings of the fly are likely to be located at the green circle.

Figure 7 displays six filtered images with different threshold ranges. The image is the same as Figure 6. Two search points are created at the end of the major axis. The boundary of each search area is drawn as a red circle and a green circle. The higher the number of positive pixels that a search point found, the higher the probability of that point to contain wings. Thus, the heading direction is to the opposite direction. According to Figure 7, wings are located at the green circle and this implies that the true head of the fly is at the red circle.
**Algorithm 1:** Heading direction detection algorithm.
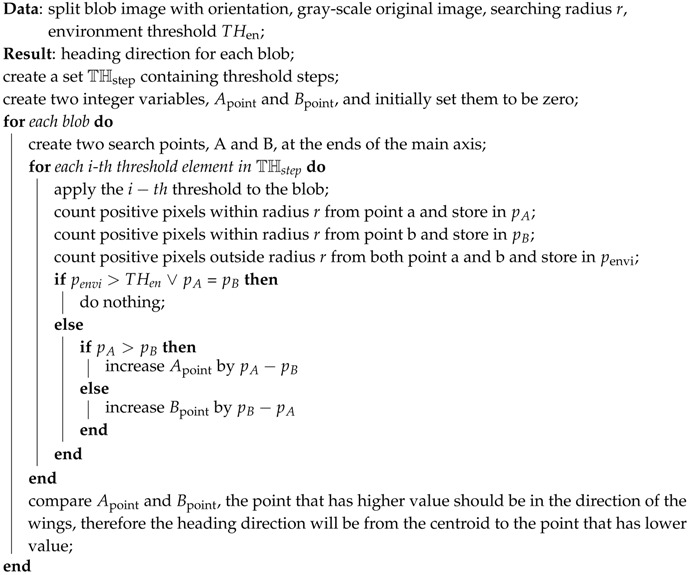


### 3.3. Tracking

Once the moving pixels or the foregrounds are detected as blobs in each frame, the next task is to assign identities to those blobs and find their correspondence in the next frame. After a fly has been assigned an identity, the identity should be kept with the same fly throughout the frame sequences, unless it may result in identity loss that will obviously affect the accuracy of further measurements. Tracking is the process of retaining the identity of each blob. There are some situations in which identity loss happens such as jumping behavior, overlapping, and crossing. These problems introduce difficulty to the tracking task and a tracking strategy that is robust to those situations is required. In our work, a Kalman Filter is used as a position and velocity predictor, together with the use of Hungarian algorithm [23], to determine the best matching of identities. To solve the Hungarian algorithm problem in MATLAB, we used an open-source function called "assignmentoptimal" by Markus Buehren [24]. The Kalman filter is also used for estimating the next state of the system. Unfortunately, the prediction from Kalman Filter alone is not enough to deal with all the situations, since the flies may suddenly move backward and this will result in tracking loss or identity swapping. Thus, we introduce a method using heading direction to help confirm the identity matching. An exactly or nearly the same heading direction of two blobs in consecutive frames increases the certainty that the two blobs are the same object. The pairs that are rejected from the confirmation of the identity matching will be treated with the closest neighbor assignment algorithm.

#### 3.3.1. Kalman Filter

Theoretically, the Kalman Filter is an estimator for the linear-quadratic problem, which is the problem of estimating the instantaneous state of a linear dynamic system perturbed by white noise and the resulting estimator is statistically optimal with respect to any quadratic function of estimation error [25]. For the next consecutive state, the filter predicts state variables based on current state variables, compares and modifies with measured information from the next state, and then gives an estimation of the next state variables with minimal error. In our work, matching between predicted states and the measurement needs a confirmation from the Hungarian assignment algorithm and heading direction before states estimation. The estimated states will be kept as states for the next time step.

State vector and state transition matrix are as follows,

(11) $$\begin{array}{c} x_{k}=\left[\begin{array}{c} x\\ y\\ v_{x}\\ v_{y} \end{array}\right],A=\left[\begin{array}{cccc} 1 & 0 & \Delta t & 0\\ 0 & 1 & 0 & \Delta t\\ 0 & 0 & 1 & 0\\ 0 & 0 & 0 & 1 \end{array}\right] \end{array}$$

where $v_{x}$ and $v_{y}$ are velocity in the *x* and *y* direction, respectively. Δt is the time difference between two consecutive frames with the state prediction equation as

(12) $$\begin{array}{c} x_{k+1}=Ax_{k} \end{array}$$

#### 3.3.2. Hungarian Assignment Algorithm

**Figure 8:**
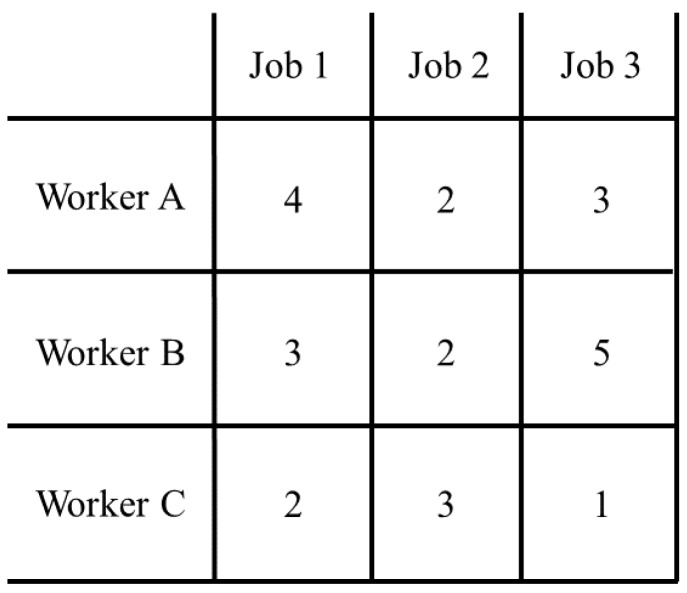
Example of a cost matrix for assigning a job to a worker. Our system assigns identities between two consecutive frames, using predicted positions from the first frame and detected positions from the second frame as the workers and the jobs. The cost will be the distance between all possible pairs of positions.

The Hungarian assignment algorithm is an optimization algorithm that solves the assignment problem. The assignment problem can be considered as assigning workers with jobs. The optimum of the assignment is achieved by giving each worker a suitable job that minimizes overall cost. An example of the cost matrix for assigning a job to a worker is shown in Figure 8. In identity assignment between two consecutive frames, the predicted result from the detection stage in the first frame is considered as the worker while detection in the second frame is considered as the job from the cost matrix in Figure 8. The cost of assigning a job to a worker is the Euclidean distance between the prediction and the detection.

#### 3.3.3. The Closest Neighbor Assignment Algorithm

The closest neighbor assignment algorithm solves the assignment problem by considering the smallest cost as the highest priority. This algorithm is different from the Hungarian assignment algorithm in the way that it focuses on the individual cost instead of the overall cost. Therefore, the result of using this algorithm does not guarantee the minimum overall cost. However, since sometimes the tracking movement is not properly estimated, such as moving forward and then backward or jumping in a different direction to the previous movement, which will result in failure of estimation, the closest neighbor assignment algorithm is then required to solve such a problem. Note that in our work, this algorithm will be used as a second assignment algorithm dealing with failed cases from heading direction, checking after the Hungarian assignment algorithm, which is the main algorithm.

The closest neighbor assignment algorithm (Algorithm 2) works by finding the minimum cost in the cost matrix. It takes the pair that has the smallest cost and puts it into a chosen pair set. The cost matrix is then modified by deleting a row and a column involving the chosen pair, thus other pairs that include elements from that extracted pair are removed from consideration. The algorithm goes back to finding the minimum cost using the new cost matrix and this process continues until all the elements in the row or the column are removed.
**Algorithm 2:** The closest neighbor assignment algorithm.
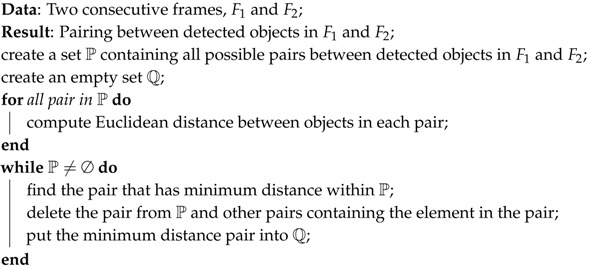


#### 3.3.4. Tracking Process

In the tracking process, there will be a tracker following the movement of each foreground object in the scene. The tracker keeps information of a foreground object such as position, velocity, and heading direction. The velocity of each detection is initially set to be zero. Next, a prediction step in the Kalman Filter is applied to all the trackers. The predicted results will be treated as the worker in the Hungarian assignment algorithm. All detection in the next consecutive frame will be input into the same assignment algorithm as the job. In this step, the assignment cost matrix is created and the cost will be the Euclidean distance between the worker and the job. Note that the number of workers and jobs may not be the same since some objects may disappear due to overlapping or miss-detection. This makes the assignment cost matrix a non rectangular matrix that may cause the Hungarian assignment algorithm in some programs to fail. Adding dummy workers or dummy jobs makes the cost matrix a rectangular matrix.

After proper matching is obtained, the heading direction of the tracker and its pair are checked in order to ensure that they are the same object. An angle between two directions tells the difference between them. If the angle lies within a specific range defined by user, the pair is preserved. If it does not, then the pair is rejected. The pair that includes a dummy will be rejected as well.

For all pairs that are rejected, a new cost matrix is created based on those pairs. This new cost matrix is different from the previous cost matrix in the way that it uses original states as the worker instead of predicted states. After the pairing step, all the information such as position, velocity, and heading direction for the next time step will be kept in each tracker. All the processes are repeated at each time step, from the desired starting frame until the desired ending frame.

**Figure 9:**
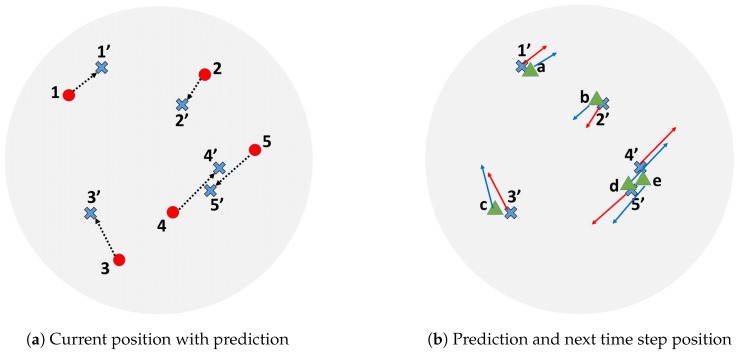
Tracking process overview. The first position of each fly in the next time step is predicted by using its current position shown in red dots and current velocity shown in the dotted vector. Next, detected positions in the next time step are paired with the predicted positions by using the Hungarian assignment algorithm. The result of pairing is confirmed by checking the heading directions. The system rejects the pair that has a high direction difference. In this figure, the pairs {4′, *e*} and {5′, *d*} will be rejected and refined with the closest neighbor assignment algorithm.

Figure 9 shows the overview of the tracking process. Suppose that the current frame is *k* (Figure 9a), trackers are labeled with number from 1 to 4 at each current position. All current positions are shown as red circles. The blue cross marks are predicted positions based on current positions and their velocities. For the next frame k+1 (Figure 9b), new positions are measured and shown as green triangles. The system then tries to pair each predicted position or blue cross mark to each measurement or green triangle by using the Hungarian assignment algorithm. There will be obvious pairs of $\left(1^{\prime },a\right)$, $\left(2^{\prime },b\right)$, and $\left(3^{\prime },c\right)$. These pairs match the state before prediction to the measurement as (1,a), (2,b), and (3,c). However, the remaining four points, $4^{\prime }$, $5^{\prime }$, *d*, and *e*, are quite problematic. If we continue without checking the heading direction, $\left(4^{\prime },e\right)$ and $\left(5^{\prime },d\right)$ will be the results from pairing. However, actually, the tracker number 4 should be paired with measurement *d* and the tracker number 5 should be paired with measurement *e* since the heading directions make more sense. To obtain proper pairing, the heading direction needs to be checked. For those that were rejected, we apply the closest neighbor between its state before prediction (red circle) and measurement (green triangle). In this way, the result will be (4,d) and (5,e).

## 4. Results and Discussion

In this section, a video showing the result of processing an example Drosophila is presented. The name of the video is “Video_1”. The example video was taken at 19 fps 827 × 818 resolution in a total of 1184 frames. There are 32 flies in a closed circular arena. All flies are not marked and do not even have their wings cut.

**Figure 10:**
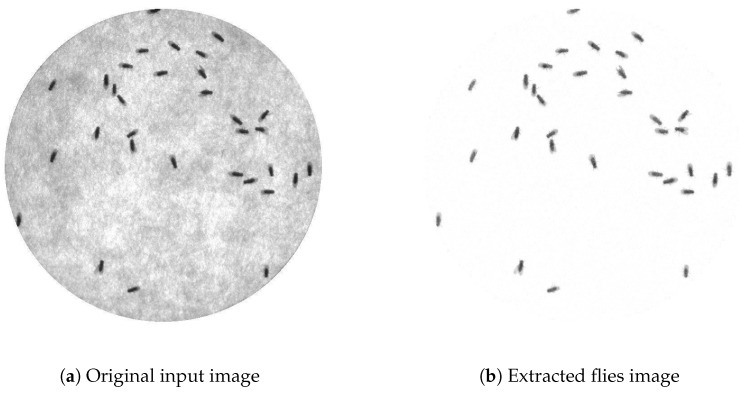
(**a**) Original input image and (**b**) extracted flies image from Video_1. There is a total of 32 files in a circular arena. The extracted flies image as a result of background subtraction followed by intensity reversal.

According to the input video, each fly appears as a dark object in the scene. The floor of the arena appears as a brighter object. Based on this statement, the proposed system detects flies using pixel intensity; a dark object will be detected as a fly. However, in some scenes, it is possible to have other objects, which are not the flies, appear in the scene, such as small tools, foods, etc. These objects appear in dark and will affect the detection process of the system. To get rid of those unwanted objects, background subtraction is performed. Figure 10 shows a grayscale input image and a scene after background subtraction. According to the figure, unwanted objects have been eliminated leaving only flies.

### 4.1. Blob Filtering and Splitting of Merging Flies

**Figure 11:**
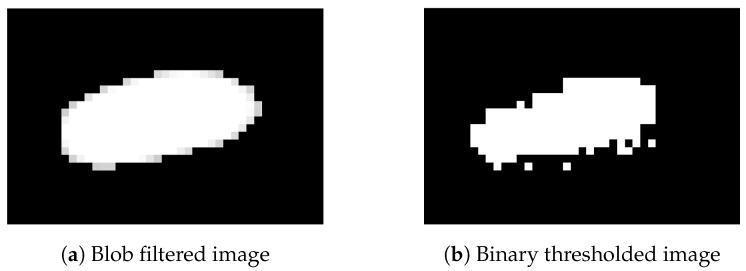
(**a**) Blob filtering and (**b**) normal binary thresholding. A comparison between applying the blob filter and applying normal binarization to a fly in the extracted flies image. The result of the blob filter provides a clear oval shape of a single fly while the result of the normal binarization contains some noises.

Figure 11 shows the processed image using the blob filter (Figure 11a) and the one using normal binarization (Figure 11b), respectively. By comparing these two figures, less noises are present in the case of using the blob filter. In this way, clearer appearance will provide a more accurate result in the posture modeling section.

Since the main situation that the system is dealing with is multiple Drosophila in a scene, there will be many situations where the flies merge. In the case of two or more flies being located close to each other, the result is merging flies detected as a single blob. Merging flies combine positions of multiple flies together, forming a new position of the group that causes the correct position of each fly to be absent. To overcome this merging problem, the system applies various intensity thresholds to the merging area in order to separate the flies. Those separated blobs will be treated in blob analysis separately, resulting in a more proper position of each fly.

### 4.2. Heading Direction

**Figure 12:**
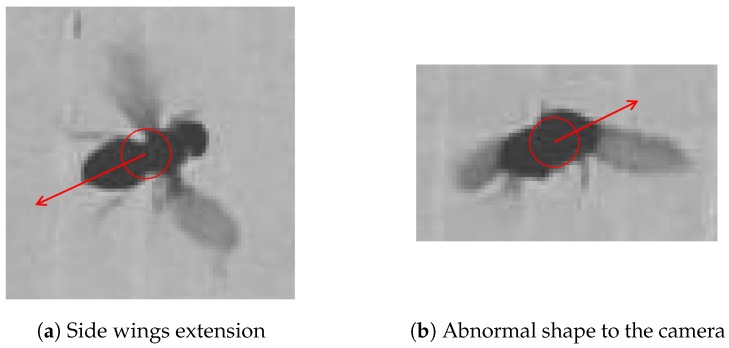
(**a**) Fly performing side wings extension and (**b**) abnormal shape detected by the camera that make heading direction fail. Having its wings in a position different from the normal wings position causes the system to fail to determine the heading direction.

Heading direction is one of the important factors that help the tracking process. There are some situations that cause error in heading direction calculation. Figure 12 shows two cases—a fly performing side wings extension and an abnormal shape—where heading direction fails. Side wings extension prevents the system from finding the position of the wings with regards to the main axis. Abnormal shape occurs when Drosophila fly and its detected heading direction becomes unknown, even using human eyes to decide.

**Figure 13:**
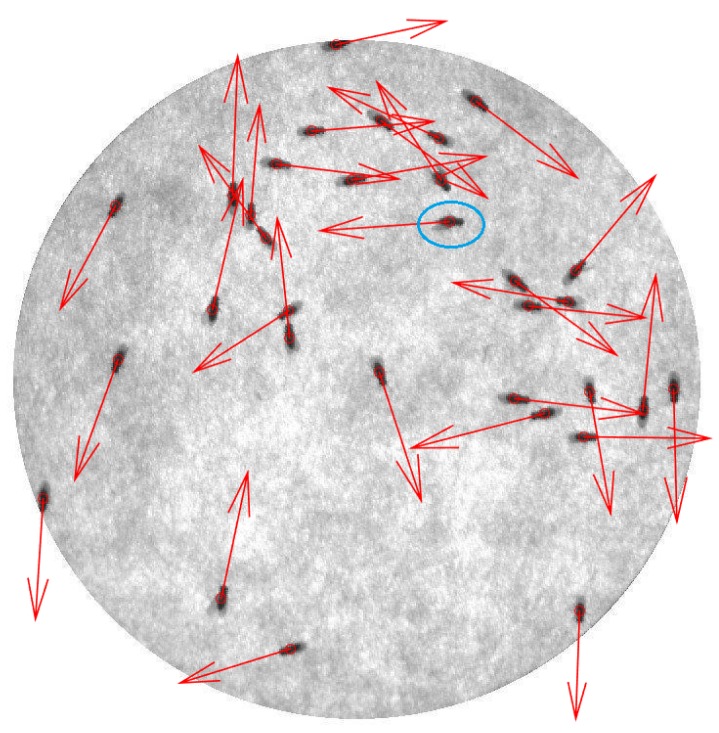
The result of determining the heading direction of each fly using Video_1. The heading direction of each fly is shown as a red arrow. Almost all heading directions were correctly determined except the fly with the blue circle.

**Figure 14:**
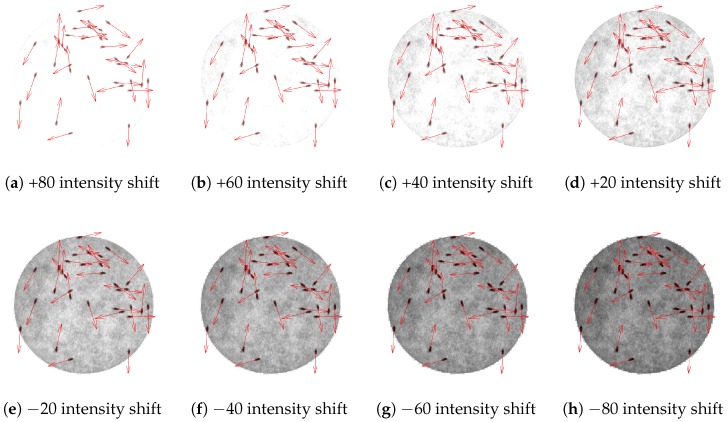
Heading direction results of various intensity shifting. The erroneous fly from Figure 13 in (**a**–**c**) appears to have the correct heading direction while (**d**–**h**) give the same result as the original scene.

Figure 13 illustrates the result of the process of determining the heading direction of each fly from “Video_1”. Heading direction is shown as a red arrow for each fly. All the information such as position and heading direction, will be kept in each frame, waiting to be used in the tracking step. The system was also tested with various intensity shifting. Figure 14 shows the heading direction result of eight intensities of Figure 13. Having the 207 mean intensity value of Figure 13 as a reference, Figure 14a–d has a mean intensity shifted by +80, +60, +40, and +20, respectively. For a negative mean intensity shift, Figure 14e–h has a mean intensity shifted by −20, −40, −60, and −80, respectively.

The system uses heading direction to help in the identity assignment step, but the heading direction does not have to be very accurate. We are currently developing the software to be able to accurately determine the heading direction.

### 4.3. Identity Assignment

**Figure 15:**
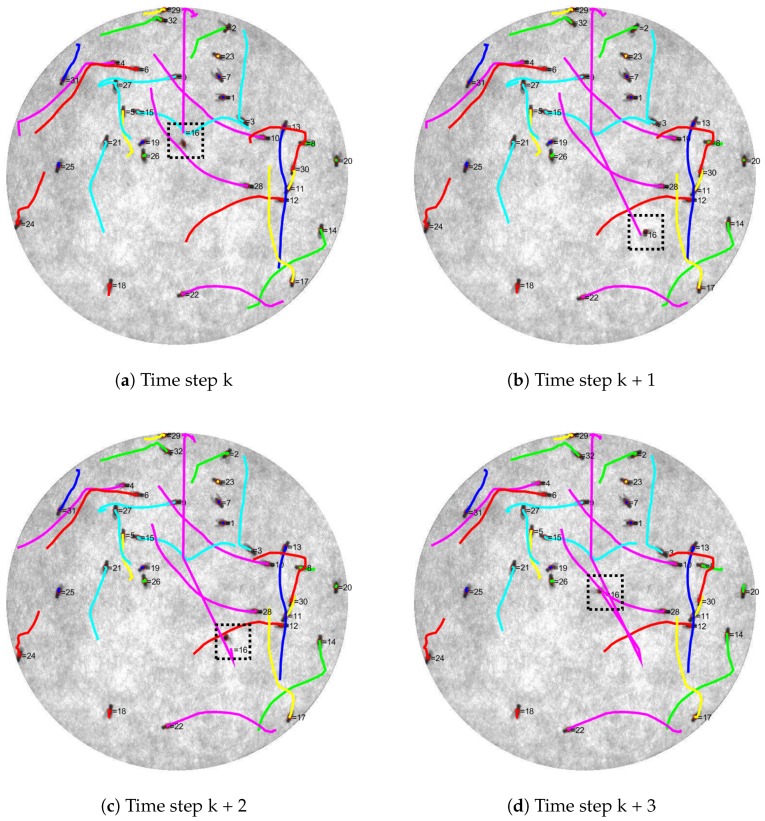
Trajectories images showing the result from the identity assignment. The colored line represents each trajectory from last six time steps. From time step k to k + 3 (**a**–**d**), there are 18 stationary flies (number 1, 2, 6, 7, 9, 12, 15, 18, 21, 23, 25, 26, 27, 28, 29, 30, and 31). Thirteen flies are detected to have small simple displacement (number 3, 4, 5, 8, 10, 11, 13, 14, 17, 20, 22, 24, and 32). There is a flying fly (fly number 16 enclosed by the black dotted rectangle). At time step k, it flew from the top of the arena to the middle and to the bottom right at time step k + 1. The next time step, it went a bit upward and finally flew back to the middle of the arena at time step k + 3. Each time step is different from its consecutive frame by 0.0526 s.

Figure 15 shows an example of tracking result. Each fly in the scene is labeled with a number corresponding to its tracker number. Let the current frame be frame *t*; the trajectory of the movement of each fly from frame t−6 to frame *t* is illustrated by a colored-line. According to the figure, all identities are assigned properly to all Drosophila. In the case of a flying fly (fly number 16 that is enclosed by the black dotted rectangle), it can also be tracked by our system.

**Figure 16:**
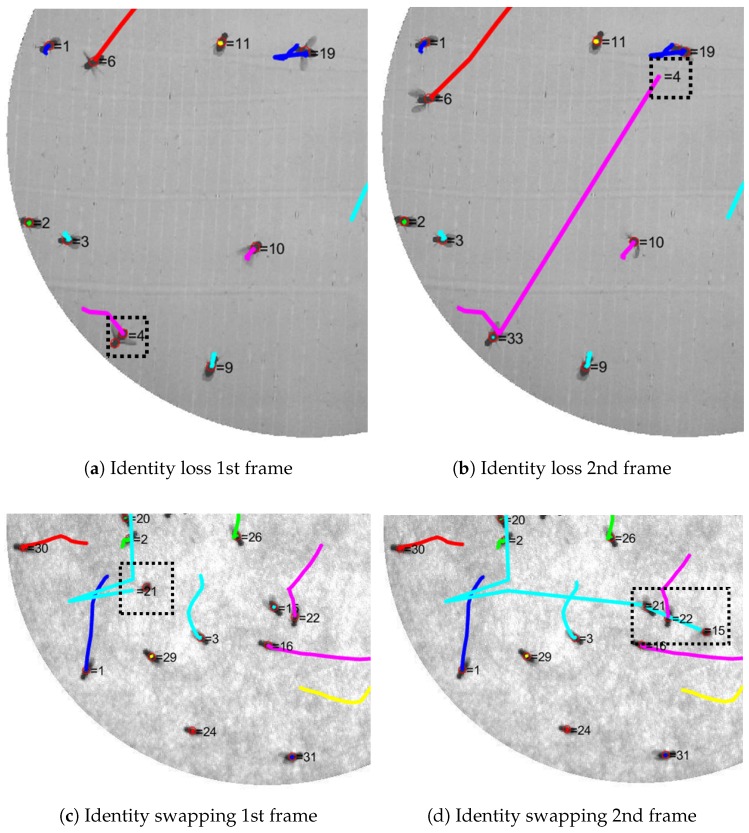
Examples of identity loss and identity swapping scenes from two different videos. Identity loss occurred at fly number 4 in (**a**) and it was lost in (**b**). The new tracker number 33 was assigned to the fly instead. Identity swapping is shown in (**c**) and (**d**). Fly number 21 was flying (**c**) and passed fly number 15 in the next frame. Their identities swapped as seen in (**d**).

Figure 16 shows examples of identity loss and identity swapping. Identity loss usually occurs when detection failed. This causes the tracker to incorrectly track the fly with noise from failed detection. Identity swapping sometimes occurs when the fly flies over another fly with a failed heading direction.

Table 1 shows the efficiency of our tracking system. There is a total of five videos shown in the table. The provided video, “Video_1”, has a resolution of 827×818 and was taken at 19 fps. Other videos (Video_2 to Video_5) were taken at 20 fps with a resolution of 593×592, 593×592, 980×980, and 1031×1031, respectively.

For Video_1 which is the main example in this part, the total number of frames is 1184 with 32 flies in a closed arena. The average velocity in each video is represented by $v_{\mathrm{av}}$. This average velocity shows how active the flies are in each video. The “Crossing” column from the table contains the number of crossing events that occurred in each video. The crossing event is an event in which two or more flies overlap, causing one detection. All errors were observed by the human eye for all frames. The swapping error was obtained from counting the number of identity swapping occurrences throughout all the frames. The number in the parentheses is the swapping error caused by the crossing event. Identity loss error is the cumulative number of losses of identity events. It counts when an identity leaves its current fly. The last column of the table represents the number of false positives (FP), the detection of non-fly objects and additional detection due to the error of blob splitting. For our tracking system, there are 10 flies swapping. There is no identity loss or FP throughout all the frames in Video_1 to Video_4. The identity loss in Video_5 occurred because of failure in the fly detection stage. It can be seen from Figure 16a that an additional detecting result was added to the fly number 4 and the identity number 4 was paired with another point in Figure 16b. This is a problem to our program since it uses a certain value of fly size to split a single blob of multiple flies. There will be no problem in a scene of multiple flies whose sizes are about the same, but it will introduce errors in a scene of flies varying in size.

Comparing with C-Trax, the proposed system still has a higher tracking error rate compared with that which is reported for C-Trax [20]. However, according to the article, the evaluation by C-Trax was done with videos that have no jumping flies. Thus, the proposed system is dealing with more extreme conditions and we are developing the system for better tracking results. Software of the proposed system was written in MATLAB programming language. The software is called “TPro”, running on Windows OS. The requirement for using Tpro is just installation of MATLAB Runtime that is a standalone set of MATLAB shared libraries. The user can define a specific range of processing frames. Only a threshold value is required from the user to set the range. The program also provides an option for the user to try various threshold values and choose a proper value to use in the fly detection step. All supplementary videos, software, and examples of the proposed Drosophila tracking system including resulting videos are also available online at [26].

## 5. Conclusions

In this paper, a system for detecting and tracking a group of Drosophila melanogaster is introduced. The system can get rid of unwanted objects besides the flies themselves. Our system can deal with situations where flies are within close proximity of one another resulting in merging of their position while having the ability to separate them apart. It also provides accurate results of posture modeling in terms of position and heading direction of each individual fly. The system uses the Kalman filter in the identity assignment and tracking part to refine the measured position and to obtain velocity. It uses predicted position along with the Hungarian assignment algorithm to get the match of each individual fly for the next frame. Moreover, the closest neighbor assignment algorithm is used for the case of heading direction mismatch, resulting in more proper matching.

Instead of doing the detection and the tracking instantaneously at each frame, the system first performs detection on all the frames, then the tracking. This makes it easy for the user to check and to refine the detection result. Furthermore, the same detection result can be used with different methods in the tracking part. This saves the user a lot of time when working with many video files.

## Figures and Tables

**Table 1:** Error of flies swapping. The first column is the video name. The second column shows the number of frames with the number of flies per frame. The third column shows average velocity. The forth column is the number of crossing events that occurred in each video. The fifth column shows the total number of swapping occurrences with the number of swaps from crossing events in parentheses. The sixth column represents the number of loss of identity events. The last column is the total number of false positives (FP).

Name	Frames (Flies/Frame)	$v_{\mathrm{av}}$ [Pixel/Second]	Crossing	Swapping (Cross-Swap)	Loss	FP
Video_1	1184 (32)	93.78	5	10 (4)	0	0
Video_2	1500 (26)	13.34	7	2 (0)	0	0
Video_3	1043 (25)	22.63	14	7 (7)	0	0
Video_4	987 (27)	37.48	16	3 (3)	0	0
Video_5	1269 (31)	34.07	16	13 (6)	4	472
